# BAFF and BAFF-Receptor in B Cell Selection and Survival

**DOI:** 10.3389/fimmu.2018.02285

**Published:** 2018-10-08

**Authors:** Cristian R. Smulski, Hermann Eibel

**Affiliations:** Faculty of Medicine, Center for Chronic Immunodeficiency, Medical Center – University of Freiburg, Freiburg, Germany

**Keywords:** BAFF - B-cell activating factor, TNFRSF13C, BAFF-R, BAFF-R deficiency, B cell, NF-k B, primary immumunodeficiencies

## Abstract

The BAFF-receptor (BAFFR) is encoded by the TNFRSF13C gene and is one of the main pro-survival receptors in B cells. Its function is impressively documented in humans by a homozygous deletion within exon 2, which leads to an almost complete block of B cell development at the stage of immature/transitional B cells. The resulting immunodeficiency is characterized by B-lymphopenia, agammaglobulinemia, and impaired humoral immune responses. However, different from mutations affecting pathway components coupled to B cell antigen receptor (BCR) signaling, BAFFR-deficient B cells can still develop into IgA-secreting plasma cells. Therefore, BAFFR deficiency in humans is characterized by very few circulating B cells, very low IgM and IgG serum concentrations but normal or high IgA levels.

## BAFFR structure and expression

Structurally, BAFFR is an atypical representative of the TNF-receptor super-family. Members of this family are typically characterized by several extracellular cysteine-rich domains (CRDs), which serve for ligand binding as well as for ligand-independent assembly of receptor monomers into dimers, trimers or multimers (1–6). Unlike most other TNF-R family members, BAFFR contains only a partial CRD which serves for ligand binding as well as for self-assembly (7).

**Figure 1:**
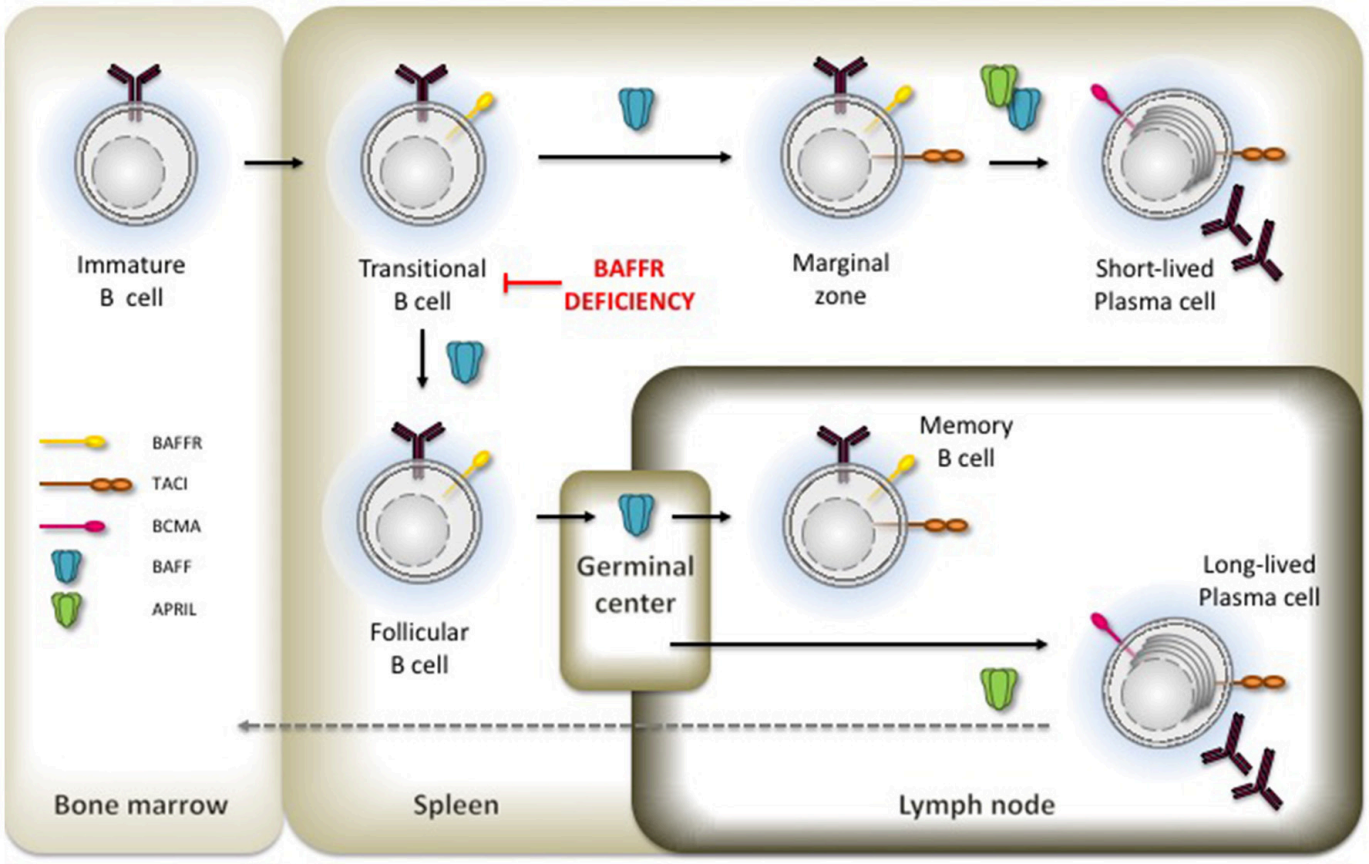
Expression of BAFFR. TACI and BCMA in B cell development. Critical developmental steps depending on BAFF and APRIL-induced signals are shown by the presence of the respective ligand.

B lymphocyte development in human bone marrow proceeds through successive stages that are defined by the immunoglobulin gene rearrangement process. After the assembly and cell surface expression of functional IgM molecules, immature IgM^+^ B cells are tested for their reactivity with self-antigens, which eventually can be corrected by receptor editing. Then, transitional B cells with low avidity to self-antigens can leave the bone marrow, enter the circulation, and migrate to the spleen where they complete these early steps of B cell development [reviewed in (8)]. BAFFR [BAFFR = *B* cell *a*ctivating *f* actor of the TNF-*f* amily *r*eceptor (9); a.k.a. BR3, Bcmd (10–12)] expression starts when the immature B cells develop to transitional B cells (13, 14), which then receive BAFFR-dependent pro-survival signals to rescue them from premature cell death (15–17). At protein level, the BAFFR is expressed on the surface of all human peripheral B cell subsets except for plasma cells and for centroblasts located in the dark zone of germinal centers. Its expression is upregulated after the expression of functional B cell antigen receptors (BCR) in response to tonic BCR signaling, which enhances BAFFR expression by immature and transitional B cells (15, 18). Although the induction of BAFFR expression seems to depend on the expression of functional B cell receptors, it can be maintained in mice after the ablation of SYK, a key element of BCR signaling (19), as well as in cells which were depleted from Ig-α (CD79A), an essential BCR component (20), supporting in both cases the survival of B cells with impaired BCR-signaling. Figure 1 summarizes the development of immature/transitional B cells and the expression of receptors for BAFF in the different subsets.

## Ligand binding

BAFFR binds the TNF-like molecule BAFF [BAFF = *B* cell activating *f* actor of the TNF-*f* amily (21); a.k.a. BLYS (22), TALL-1 (23), zTNF4(24)] as single ligand (9, 12, 25). In contrast, the two other closely related, BAFF-binding receptors TACI (*T* cell *a*ctivator and *c*alcium modulating ligand *i*nteractor) and BCMA (*B c*ell *m*aturation *a*ntigen) (24) can additionally bind to APRIL [*a pr*oliferation-*i*nducing *l*igand, (26)], another member of the TNF-α family, which supports the survival of plasma cells (27). TACI is expressed by activated B cells, marginal zone B cells, switched memory B cells and by plasma cells (24, 28–30). Compared to BAFFR, TACI has different functions by serving on the one hand as decoy receptor (31) while triggering, on the other hand, immunoglobulin class-switch recombination (32). Different from BAFFR and TACI, BCMA is upregulated in activated B cells and expressed constitutively by long-lived plasma cells supporting their survival (33). Both ligands, BAFF (21, 22) and APRIL (26) are type II transmembrane proteins forming homo- as well as heterotrimers (22, 34–36). BAFF is expressed as membrane-bound ligand (see also contribution of Kowalczyk-Quintas et al. to this Research Topic), which is then processed by the membrane-bound protease furin resulting in a soluble form. Soluble BAFF exists as trimer and can associate into a virus-like capsid called 60-mer, composed of 20 trimeric units (35, 37, 38). The assembly of 60-mers depends on trimer-trimer interactions mediated by a small loop called the “flap” (34, 35). Without the flap region BAFF can still assemble into trimers, which can bind to BAFFR but this form of BAFF does neither initiate downstream signaling cascades nor does it support B cell survival. Therefore, crosslinking of multiple BAFF-BAFFR complexes via the flap-region is essential for BAFFR-dependent B cell responses (39).

BAFF and APRIL are expressed by monocytes, macrophages, dendritic cells, bone marrow stroma cells (21, 23, 40), and by T cells (21, 41). The expression of both ligands increases under pro-inflammatory conditions (42) and correlates inversely with the expression of BAFFR and TACI (43).

## BAFFR signaling and target genes

BAFF binding to BAFFR activates several downstream pathways that regulate basic survival functions including protein synthesis and energy metabolism required to extend the half-life of immature, transitional, and mature B cells.

Like the TNF-receptor family members CD40, LTβR, and RANK, BAFFR triggers the non-canonical NF-κB2-dependent pathway (44, 45). NF-κB2 belongs together with NF-κB1, RelA, RelB, and c-Rel to the group of NF-κB/Rel transcription factors [reviewed in (46)]. Activation of NF-κB1 is a very rapid process (47). Upon MAP3K7 (TAK1)-dependent phosphorylation, IKKα, IKKβ, IKKγ assemble into the inhibitor of kappa-B kinase (IKK) complex and phosphorylate IkBα, the inhibitor of NF-κB1. This allows its proteasomal degradation and nuclear translocation of NF-κB p50/relA and p50-c-rel heterodimers, which are constantly generated by the association between the processed form of NF-κB1/p105 and relA or c-Rel. In the nucleus, the NF-κB heterodimers mainly act as transcriptional activators regulating a plethora of target genes including the gene for NF-κB2 (48).

Different from NF-κB1, the activation of the non-canonical NF-κB2 pathway by BAFF is a slow and complex process (44) which relies on the activation of the NF-κB inducing kinase NIK (MAP3K14) (49, 50). In the absence of BAFF (or of other NF-κB2-inducing factors), NIK binds TRAF3, which promotes the proteasomal degradation of the kinase after its ubiquitinylation by a complex with ubiquitin-E3 ligase activity composed of the cellular inhibitors of apoptosis cIAP1 and cIAP2, TRAF2 and TRAF3 (51). NIK degradation prevents the accumulation of the kinase and the phosphorylation of NF-κB2 by IKKα (IKK1), which itself is activated through phosphorylation by NIK (52). When BAFF binds to BAFFR, it leads to the aggregation of BAFF receptors which recruit TRAF3 to their intracellular part. This allows the dissociation of the NIK-TRAF2/3-cIAP1/2 complex (53–55), exposes the TRAF3 lysine residue K46 to ubiquitin ligases and allows proteasomal degradation of TRAF3 (56). By reducing the number of available TRAF3 molecules, newly synthesized NIK can accumulate (53) and phosphorylate IKK1 (52). The active form of IKK1 then phosphorylates NF-κB2 p100 at the C-terminal serine residues 866 and 870 (50), which now becomes a target of the E3 ubiquitin ligase βTrCP, which adds ubiquitin to lysine residue K856. Ubiquitinylated NF-κB2 then binds to the regulatory subunit of the proteasome (56) promoting the cleavage of the p100 precursor into the active p52 form which forms an heterodimer with relB and translocates into the nucleus to regulate the transcription of NF-κB2 target genes. The complexity of this multi-step reaction, which is dependent on the relative concentration of TRAF and its association with BAFFR (57) explains why BAFF-induced activation of NF-κB2 target genes is a slow process.

Downstream of NF-κB2 several target genes have been identified. These include ICOSL, a co-stimulatory ligand for ICOS, which is expressed by activated T cells, provides co-stimulatory signals, and promotes the development of follicular T-helper cells (58). Analysis of B cells from NIK-deficient patients underlines the role of NIK and NF-κB2 in activating ICOSL expression as NIK-deficient cells do not upregulate ICOSL in response to CD40L (59).

As pointed out above, BAFF binding to BAFFR recruits TRAF3 to the receptor reducing the concentrations of available TRAF3. This allows—similar to the accumulation of NIK—the accumulation of the transcriptional regulator “cAMP response element binding protein” CREB, which in resting cells is initially complexed to TRAF3 inducing its ubiquitinylation and degradation. Accumulation of CREB increases the expression of its target gene Mcl-1 (60), a Bcl-family member with powerful anti-apoptotic activity, which acts by stabilizing the mitochondrial outer membrane (61). The deubiquitinating enzyme OTUD7B represents another NF-κB2 target gene (62). Activation of BAFFR increases OTUD7B expression and the newly synthesized deubiquitinase then binds to TRAF3 resulting in the formation of a high molecular weight complex including OTUDB7, TRAF3, TRAF2, and cIAP1/2. This leads to the deubiqutinylation and stabilization of TRAF3, which now can bind and inactivate NIK again resulting in the downregulation of BAFFR signaling. Thus, OTUB7 acts as negative regulator of the NF-κB2 and limits BAFFR-dependent cellular activation (62).

**Figure 2:**
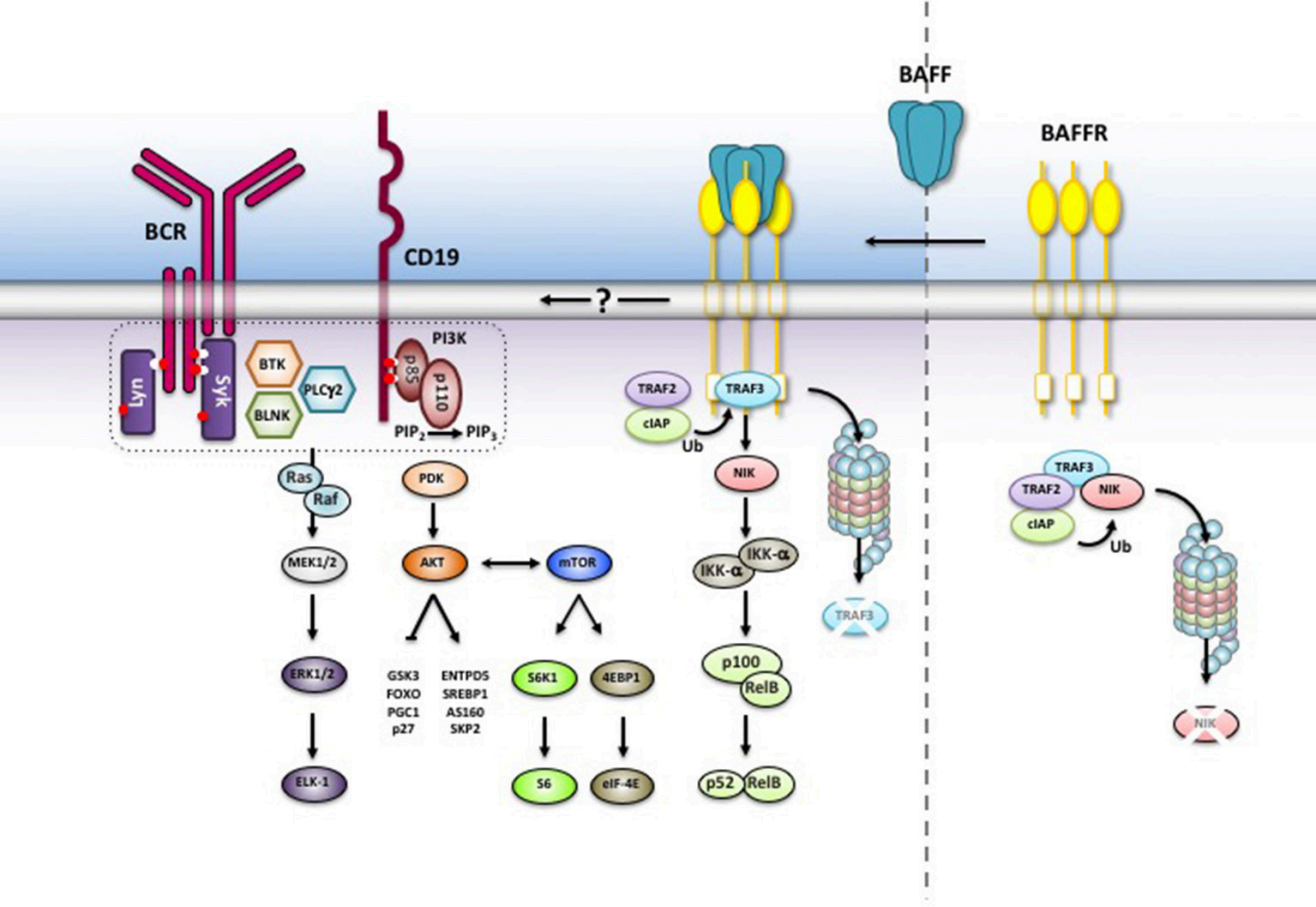
BAFFR-induced intracellular signaling. Without BAFF, NIK is complexed to TRAF3 and degraded in the proteasome. BAFF binding to BAFFR recruits TRAF3 to BAFFR and stabilizes NIK while TRAF3 is degraded by the proteasome. NIK activates the non-canonical NF-κB2 signaling pathway and allows nuclear translocation of NF-κB2 p52/relB heterodimers. BAFF binding also activates the PI3K pathway, which shares common components with BCR signaling. The exact mechanisms leading to PI3K activation are still not understood.

In addition to NF-κB2 signaling, binding of BAFF to BAFFR activates the phosphoinositide-3-kinase-dependent signaling cascade. An elegant series of experiments carried out in mice demonstrated that activation PI3K pathway by BAFFR makes use of components belonging to the B cell antigen receptor pathway (63, 64). Similar to the B cell receptor (65), BAFFR-induced signals remodel the cytoskeleton by interacting with a network which includes the tetraspanin CD81, the co-receptor CD19 and the Wiscott-Aldrich syndrome interacting protein WIP (66). This suggests that BAFFR seems to be part of a large complex of transmembrane and membrane-associated proteins using common signaling components that are activated in a context-dependent manner. Downstream of PI3K, the AKT/mTOR axis initiates the metabolic reprogramming of B cells resulting in an increased cellular fitness and lifespan [reviewed in (67)]. In mice, BAFF induces the PI3K-depenent phosphorylation of AKT at both Ser437 and T308 (68, 69) allowing the phosphorylation of downstream substrates including GSK3β, the transcription factor FOXO, the small ribosomal subunit protein S6 and the translation inhibitor 4EBP1. Since phosphorylated S6 activates while non-phosphorylated 4EBP1 inhibits translation, BAFFR is an important regulator of protein synthesis. In addition to protein synthesis, BAFFR-dependent activation of the PI3K pathway stabilizes MCL-1 by phosphorylating GSK3β and PIM2 (61, 68, 69), leading to enhanced mitochondrial function and an increase in ATP production. Figure 2 summarizes essential features of BAFFR signaling.

## BAFFR processing

Besides the activation of several downstream signaling pathways BAFF binding to BAFFR causes the shedding of the extracellular part of BAFFR (70). The proteolytic cleavage of BAFFR is catalyzed by the metalloprotease ADAM10 and requires the co-expression of TACI. Since TACI is expressed by marginal zone and by switched memory B cells, BAFFR shedding by ADAM10 in response to BAFF binding affects mainly these B cell subsets. After the extracellular part of BAFFR has been released, its transmembrane part and the cytoplasmic domain are internalized and translocated to lysosomes where they are most likely degraded. Thus, BAFFR processing differs from the shedding of TACI, which is cleaved constitutively by ADAM10 in the absence of ligand. Processing of TACI removes its extracellular domain, which can act as decoy receptor for both BAFF and APRIL (31). Similar to TACI, BCMA is also processed constitutively, not by ADAM proteases but by γ-secretase (71). As it has been described for Notch, γ-secretase also cleaves the part of TACI which remains in the plasma membrane after the extracellular part has been shed by ADAM10 (31), but it remains to be shown if the released intracellular form of TACI has a biological function analogous to the intracellular part of Notch, which participates directly in the transcriptional regulation of target genes (72).

Since BAFFR shedding is induced by ligand binding to BAFFR, the released extracellular domain of BAFFR is most likely still complexed with BAFF preventing its function as soluble decoy receptor like soluble TACI or BCMA. If BAFF dissociates from soluble BAFFR, the soluble form of BAFFR most likely also looses its ligand binding activity, which also argues against its decoy receptor function. As a result of the constitutive shedding of TACI and BCMA, the surface expression levels of both receptors are rather low (31, 71), and differs from BAFFR expression levels, which are high on all B cell subsets except for germinal center B cells (70).

Germinal center B cells undergo proliferation, somatic hypermutation and immunoglobulin class-switch recombination in the dark zone, from where they migrate to the light zone, where they are selected by their affinity to their cognate antigen into the switched memory B cell and long-lived plasma cell pool (73). While light zone B cells express close to normal levels of BAFFRs which are not bound to BAFF, BAFFRs expressed by dark zone B cells are heavily loaded with BAFF inducing BAFFR-dependent survival signals as well as BAFFR processing by ADAM17. Thus, while BAFF-induced BAFFR processing limits initial survival signals for dark zone cells (70), the survival of light zone B cells is regulated by the affinity of their surface immunoglobulins, which has to be above the thresholds set by interacting T-follicular helper cells and by the competing IgG and IgA antibodies secreted by plasma cells surrounding the B cell follicle (74).

## BAFFR and B cell survival

From the analysis of the BAFFR encoding *Bcmd* mutation discovered in the A/WySnJ mouse strain (10, 11) and after the identification of BAFF as pro-survival cytokine for B cells (21, 75) it became clear that both proteins form a ligand-receptor pair which is essential for B cell survival (9, 12). Of interest, the different mouse models revealed that not all B cell subsets are equally dependent on BAFFR-induced survival signals. While *Baffr*-deficient A/WySnJ or Baff- and Baffr-KO mice had much less follicular and marginal zone B cells (B2 B cells) than the corresponding controls, the inactivation of the *Baff* or *Baffr* genes did not affect the population of peritoneal B1 B cells (11, 25, 76). In the mouse, B1 cells form a distinct, innate-like B cell subset, which develops before and shortly after birth and is maintained by self-renewal through limited proliferation but not, as follicular and marginal zone B cells, by *de novo* generation from hematopoietic precursor cells [reviewed in (77, 78)]. Apart from differences in CD5 expression, B1 B cells can be separated into two subsets by the expression of plasma cell alloantigen (PC1; a.k.a ectonucleotide pyrophosphatase phosphodiesterase 1; ENPP1). PC1^low^ B1 cells develop from early B1 precursor cells during fetal life and differentiate in the gut into IgA secreting plasma cells (79). Interestingly, *Baff*- and *Baffr*-deficient mice as well as *BAFFR*-deficient humans have decreased titers of serum IgG and IgM but not of IgA (11, 25, 76, 80), which may be due to the differentiation of BAFF-independent PC1^low^ B1 cells developing in the gut into IgA secreting plasma cells. Similar to genetic ablation of BAFF and BAFFR expression, injection of anti-BAFF (81) or anti-BAFFR antibodies (82) or of TACI-Ig (83, 84) resulted in the depletion of transitional-2, follicular and of marginal zone B cells but spared pro- and pre-B cells, immature, and transitional-1 B cells. In addition, these depletion experiments showed that switched memory B cells and plasma cells can survive after depleting BAFF or blocking BAFF-BAFFR interactions. Thus, the development and maintenance of the follicular and marginal zone B cell pool strongly depends on BAFF and BAFFR, whereas B1 B cells and switched memory B cells can survive without BAFF, although they express similar levels of BAFFR as the BAFF-dependent subsets. Future studies have to reveal, why BAFFR is expressed on these cells and if it has any function.

## The role of BAFFR in B cell selection and autoimmunity

BAFFR expression starts in the bone marrow when B cells become IgM^+^ immature B cells, which undergo negative selection for autoreactive B cells. Since BAFFR-dependent signals prolong the survival of B cells, it was of interest to analyze if BAFF binding to BAFFR plays a role in the selection of B cells into the pool of mature B lymphocytes. Using a rearranged immunoglobulin V-gene knock-in mouse model (18), R. Pelanda and her group demonstrated that IgM^+^ IgD^−^ autoreactive B cells with low IgM surface expression express low levels of BAFFR and do not respond to BAFF-induced survival signals. In contrast, non-autoreactive IgM^+^ IgD^−^ B cells expressing more IgM on the cell surface also expressed, proportional to IgM, more BAFFR and developed into IgM^+^ IgD^+^ CD21^+^ CD23^−^ transitional-1 B cells. These cells respond to BAFFR by developing into IgM^+^ IgD^+^ CD21^+^ CD23^+^ transitional-2 cells and later into mature B lymphocytes as it had been shown before in other mouse models (76, 81). A similar conclusion was reached by the group of Rolink (85) by carefully analyzing RAG-2 expression and B cell receptor editing by mouse and human IgM^+^ immature B cells from bone marrow. They found that BAFFR surface levels correlate directly with IgM but inversely with RAG-2 expression and receptor editing. Interestingly, anti-IgM treatment downregulates BAFFR on the surface of immature and transitional B cells while it enhances its expression on the surface of mature B cells (85). Since the inactivation of two Rho-GTPase encoding genes *Rac1* and *Rac2* does not only abolish BCR-induced intracellular calcium flux and the activation of the PI3K pathway but also BAFFR expression (86), BCR-dependent activation of Rac GTPases seems to induce the transcription of the *Baffr* gene in immature B cells.

B cells undergo a second phase of selection in germinal centers. Since excess of BAFF promotes the development of autoreactive B cells (75), BAFF-induces signals which interfere with mechanisms regulating the selection of B cells in the germinal center and with the equilibrium between BAFF-induced survival of dark zone B cells and affinity-based selection of centrocytes in the light zone. Genome-wide genetic association studies carried out with samples from multiple sclerosis (MS) and systemic lupus erythematosus (SLE) patients now provide evidence that genetically encoded changes of BAFF levels result in increased concentrations and correlate with the increased risk of developing autoimmunity (87).The genetic change results from a small deletion within the 3'UTR of BAFF mRNA. The deletion creates a new polyadenylation site allowing the premature termination of BAFF transcription. This shorter version of BAFF mRNA lacks an important regulatory sequence containing the binding site for miRNA-15a. This prevents micro-RNA directed control of excessive BAFF mRNA resulting in 1.5 to 2-fold increase in BAFF levels in a gene-dosage dependent manner. Like in the BAFF-transgenic mice, higher BAFF levels in humans increase the numbers of circulating B cells, promote the development of plasma cells, and result in higher serum IgG and IgM concentrations in homozygous carriers of this *TNFSF13B* variant (87).

Ablation of TACI expression or function not only cause immunodeficiency but also increases the risk of developing autoimmunity (88–90). The autoimmunity is now best explained by the decoy receptor function of TACI. In humans, the TACI variants C104R or C104Y, which reside in the second CRD abolish ligand-binding activity of TACI without preventing cell surface expression of the receptor. ADAM10-induced processing therefore sheds soluble forms of TACI, which cannot serve as decoy receptors to neutralize excessive BAFF levels. Therefore BAFF levels are increased in TACI-deficient patients (43) enhancing the risk of developing autoimmunity and lymphoproliferation, two characteristic features described in TACI deficiency in humans (89, 90) and mice (12, 88, 91).

However, point mutations or ablation of TACI expression also causes immunodeficiency. This can be best explained by the role of TACI in supporting T-independent immune responses (32, 92–95) and the survival of plasma cells (28, 30).

## BAFFR deficiency in humans

In humans, only two cases of BAFFR-deficiency resulting from complete inactivation of the BAFFR encoding gene *TNFRSF13C* have been described so far. In both cases, the autosomal-recessive, homozygous 24bp in-frame deletion (80) removes the codons of highly conserved eight amino acids (LVLALVLV) from the transmembrane region of BAFFR, which extends from residues (76–98). The truncated BAFFR protein is highly unstable although *in silico* modeling predicts that the mutant BAFFR protein would be able to form a new transmembrane region between the resulting residues (70–92), which partially overlaps the TM region of the *wild type* protein. The lack of BAFFR expression causes an arrest of B cell differentiation at the transition from CD10^+^ immature/transitional 1 B cells to transitional 2 / naïve and marginal zone B cells. The homozygous mutation has full penetrance whereas the heterozygous deletion is phenotypically indistinguishable from healthy donors. Many of the immunological characteristics of human BAFFR deficiency have been described in Baffr-/- mice. The common features include very low numbers of circulating B cells, low IgM and IgG antibody titers but increased levels of serum IgA. Nevertheless, there are some clear differences between BAFFR-deficient humans and mice. First of all, inactivation of the BAFFR encoding *Tnfsf13c* gene in mice still allows normal development of B1 B cells, whereas in BAFFR-deficient humans have neither B1 B cells nor any other B cell subset, which would resemble B1 B cells. However, if B1 B cells would develop in humans as in mice from precursor cells during embryonic life and persist by homeostatic proliferation (78, 96, 97), they might have been disappeared in the BAFFR deficient patients like they also can disappear in old mice. On the other hand, the severe block in the development of follicular and marginal zone B cells might have created more space for the expansion of B1 B cells which then also would have had the chance to compensate the lack of these B2 B cell populations and to develop into IgA secreting plasma cells in the gut as it has been observed in mice (79). Interestingly, both BAFFR deficient patients have an effective output of immature B cells from bone marrow resulting in high numbers of transitional B cells, which are comparable to much younger adults. Different from Baffr-/- mice, both BAFFR-deficient humans suffered from severe lymphopenia. This difference might also be age-related or it might depend on differences regulating the size of the B cell pool, which are not well understood but known to vary between different individuals (98–100). Since IgM^+^ CD27^+^ marginal zone B cells control infections with encapsulated bacteria (101), the absence of marginal zone B cells resulted in defective T-independent vaccination responses against Pneumovax, which consists of a mixture of 23 different *S.pneumoniae* cell wall polysaccharides. Notably, the low but detectable IgG response against the T-dependent antigen tetanus toxoid, the high IgA serum concentrations and the presence of IgA^+^ plasma cells in the lamina propria of the small intestine showed that BAFFR-deficiency does not completely exclude the development of B cells into plasma cells. Similar results were observed in in Baffr^−/−^ mice in which BAFFR-deficient B cells were found to complete the germinal center reaction (102) and to develop into switched memory B cells and plasma cells which survive without BAFFR (103). Upon depletion of BAFF by BAFF-neutralizing treatment with anti-BAFF monoclonal antibodies (Belimumab, Benlysta) or with soluble TACI-Ig (Atacicept), BAFFR-independent long-term survival of memory B cells has also been detected in SLE patients. These clinical studies show the strong (>75%) decline of naive B cell numbers that is followed by an increase in switched memory B cells (104, 105). Similar to the numbers of switched memory B cells, IgG antibody concentrations, which were build-up before starting the BAFF-neutralizing therapy, remained constant whereas the increase of antibody titers against neoantigens from influenza virus was significantly lower in belimumab-treated patients than in controls (106). In a similar study, the population of switched memory B cells did also not decrease within a half year treatment of rheumatoid arthritis patients with TACI-Ig fusion protein atacicept (107).

In addition to the BAFFR deletion, different missense mutations have been described for BAFFR. The mutants were found in patients suffering from common variable immunodeficiency (CVID), the most frequent form of primary immunodeficiency which is characterized by low or absent IgM, IgG, and IgA serum titers, low numbers or absent circulating switched memory B cells and the absence of circulating plasma cells (108, 109). The BAFFR missense mutations change amino acid residues in the extra- or intracellular part of BAFFR (110–112) but they do interfere with B cell development or survival in a way which would be comparable to the BAFFR deletion mutant. Therefore, their contribution to the development of CVID and antibody deficiency remains to be shown. In this context, the P21R BAFFR variant, which is encoded by a frequent single nucleotide polymorphism (rs77874543), represents one exception. The proline 21 is located in a small loop directly preceding the BAFF binding domain. Functional and biochemical studies showed that this small loop region is essential for ligand-independent association of BAFFR polypeptide chains into multimers. It therefore represents the pre-ligand assembly domain of BAFFR (7). Although the P21R-related defect in BAFFR clustering reduces the number of BAFF molecules able to bind BAFFR on the surface of B cells by at least 50%, it does not interfere with the development of transitional B cells to naive mature B cells. Since BAFFR multimerization strongly enhances BAFF binding, B cells carrying the P21R mutation develop less efficiently into IgM secreting plasmablasts. Moreover, the homozygous P21R variant is completely resistant against BAFF-induced processing of BAFFR by ADAM10 (70): The mutation seems to compensate its reduced ability to bind BAFF. This feature may mask in part the impaired differentiation of P21R^+^ B cells into IgM secreting plasmablasts and prevent the development of overt immunodeficiency. Table 1 summarizes the main differences in the studies of BAFF and BAFFR in humans and mouse models.

**Table 1:** Comparison between human and mouse BAFFR.

**BAFFR**	**Human**	**Mouse**
Expression starts in immature IgM^+^ D^−^ bone marrow B cells	+	+
Low expression during receptor editing	+	+
Expression induced by BCR signaling	+	+
Supports survival of transitional, follicular and marginal zone B cell	+	+
BAFFR-independent survival of switched memory and plasma cells	+	+
BAFFR-independent survival of B1 B cells	B1 B cells not found	+
High IgA levels in BAFFR deficiency	+	+
BAFF-induced BAFFR processing by ADAM10	+	Not analyzed
ADAM17-dependent BAFFR processing in dark zone GC B cells	+	Not analyzed
BAFFR-induced NIK-dependent activation of NF- κ B2	+	+
BAFFR-induced activation of PI3K	+ (B lymphoma cells)	+
BAFFR-induced activation of ERK	+ (B lymphoma cells)	+
Autoimmunity induced by high BAFF concentrations	Genetic association with SLE and MS	+

## Perspectives and conclusions

Studying the roles of BAFF and BAFF-receptors in experimental systems allowed the development of drugs which are now used to treat autoimmunity. So far, the BAFF-neutralizing monoclonal antibody belimumab is the only FDA- and EMA-approved biological drug for the treatment of patients with active refractory SLE. Application of the anti-BAFF antibody leads to a persistent, 50% improvement in about half of the SLE patients as reflected by lower anti-dsDNA titers, less corticosteroids, less cutaneous manifestations and less flares, while its adverse effects are similar as but less severe than the defects observed in BAFFR-deficiency and include respiratory, digestive and urinary tract infections [reviewed in (113)]. However, because of the lack of matched control groups, the efficacy of belimumab is still not completely clear. A second anti-BAFF antibody (tabalumab or LY2127399) has been tested in phase III studies and had similar effects like belimumab with an increase in complement activity and a decrease of anti-dsDNA IgG titers and B cell numbers leading to a mild clinical improvement in about 35% of patients treated in response to high dose treatment (114). The combination of tabalumab and bortezomib in a phase II study with multiple myeloma patients did not improve progression-free survival of the patients indicating that BAFF plays little or no role in disease progression (115). Similar to SLE, hyperactivated B cells are also discussed to play an important pathological role in the Sjögren's syndrome (116). Although in a trial study the treatment with the B cell-depleting anti-CD20 antibody did not lead to any improvement (117), new clinical trials with a novel BAFFR-specific monoclonal (ianalumab/VAY736/NOV-5) just have started. Since increased BAFF levels correlate with the risk of developing multiple sclerosis (87), new clinical trials with MS patients with tabalumab are now being performed (118). However, it should be kept in mind that treatment of MS patients with atacicept resulted in severe adverse effects, suggesting that in MS the blockade of BAFF and APRIL removes B cells with regulatory and immunosuppressive function and spares the pathological cells (119, 120).

In summary, switched memory B cells can perfectly survive without receiving BAFFR- or TACI-dependent pro-survival signals and develop into IgA secreting plasma cells. Different from B1 B cells and from switched memory B cells, the survival of transitional, follicular and marginal zone B cells as well as the differentiation of transitional B cells into follicular and marginal zone B cells depends essentially on BAFFR-induced survival signals, which increase the life span of these cells by stabilizing mitochondria and by enhancing protein synthesis. Since IgA-secreting plasma cells can develop even in the absence of BAFFR, BAFFR-deficiency does not become manifest as dramatically as NIK or NF-κB2 deficiency, which both strongly impair B and T cell responses. Since BAFFR, TACI and BCMA play different but critical roles in regulating B cell development and survival, analysis of coupled signaling pathways, of processing reactions affecting the half-life of surface BAFFR, TACI and BCMA and of their protein interaction partners will provide deep insights into the mechanisms regulating B cell selection, autoimmunity and aging.

## Author contributions

All authors listed have made a substantial, direct and intellectual contribution to the work, and approved it for publication.

### Conflict of interest statement

The authors declare that the research was conducted in the absence of any commercial or financial relationships that could be construed as a potential conflict of interest.
